# Abscopal Effect of Radiotherapy in Imatinib-resistant Dermatofibrosarcoma Protuberans

**DOI:** 10.7759/cureus.3857

**Published:** 2019-01-08

**Authors:** Mervin B Agyeman, Verna D Vanderpuye, Joel Yarney

**Affiliations:** 1 Radiation Oncology, National Radiotherapy Oncology and Nuclear Medicine Centre, Korle-Bu Teaching Hospital, Accra, GHA

**Keywords:** abscopal, radiation, imatinib

## Abstract

Local tumor control and symptom relief have been the major advantage of radiotherapy in clinical practice. In the past years, the systemic anti-tumor effect of radiotherapy, also known as the abscopal effect, has been reported with limited studies. With the advent of immunotherapy, the frequency of the abscopal effect has increased in patients who receive sequential treatment with radiotherapy and immunotherapy or patients who receive radiotherapy after acquiring resistance to immunotherapy. A novel cancer treatment modality, such as molecular targeted therapy, has been associated with the immune response within the tumor but its systemic anti-tumor effect, when combined with radiotherapy, is yet to be documented. There have been few studies to date assessing the immunological effects of imatinib on tumors; however, the mechanism of tumor regression or resistance acquisition is poorly understood. We present a 56-year-old male diagnosed with dermatofibrosarcoma protuberans (DFSP) who developed resistance to imatinib after five months of treatment. Following subsequent local radiotherapy to the primary tumor, he had complete clinical remission of the primary and metastatic lesions.

## Introduction

The concept of achieving “out-of-target” tumor regression with localized radiotherapy, the abscopal effect, was initially reported in 1953 [1]. Further reports confirmed the effect in distant normal tissue, resulting in lymphopenia and impaired leukocyte function, which contribute to the immunosuppressive effect of radiotherapy [2]. The exact mechanism of the abscopal effect is poorly understood. Over the years, a few sporadic cases have been documented; the majority of which occurred in immunogenic tumors such as renal cell carcinoma, melanoma, and hepatocellular carcinoma [3]. With the advancement of cancer research, the immune system has been hypothesized to play a major role in eliciting the abscopal effect [4]. This has paved the way for immunomodulators and the immune checkpoint blockade to be used with radiotherapy to achieve tumor regression even in less immunogenic tumors such as breast carcinoma [5]. Targeted therapy has gained a significant role in cancer management, with milestones achieved through research. Immune-mediated effects have been noticed with therapies such as imatinib [6]. Concurrent imatinib with radiotherapy has been used to achieve a desirable outcome at a lower radiation dose in desmoid tumors [7], but its systemic anti-tumor effect is yet to be observed. Despite the advantages the abscopal effect may have on overall survival and disease-free progression, its relevance in current radiation treatment protocols remains uncertain because of the paucity of research in that area [3]. We present a case report showing the positive impact of the abscopal phenomenon.

## Case presentation

A 56-year-old male presented to a hospital in Togo, West Africa, with a long-standing swelling on the left lower leg, which progressively increased in size over time. He had a wide local excision done following a confirmatory biopsy for dermatofibrosarcoma protuberans (DFSP). No adjuvant therapy was recommended on account of clear surgical margins and the absence of distant metastases. The lesion recurred after two years and re-excision was performed. A second recurrence occurred in a year, which involved the knee joint, necessitating a transfemoral amputation. The surgical margin was clear and there was no evidence of distance metastases. He was rehabilitated and started walking with a prosthetic limb.

**Figure 1:**
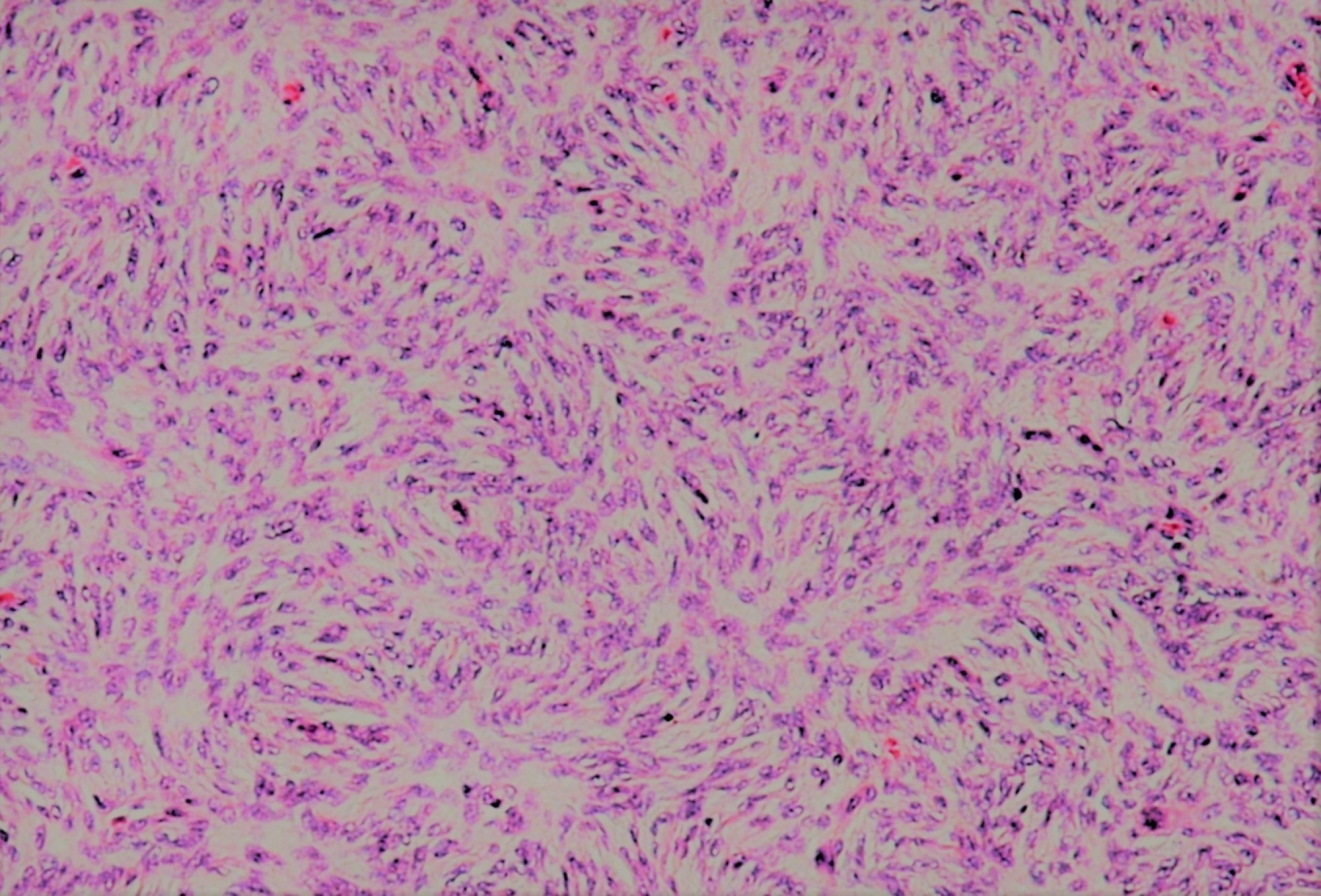
Microscopic pathology image showing the storiform pattern with a low to moderate nuclear mitotic rate (hematoxylin and eosin stained tissue)

**Figure 2:**
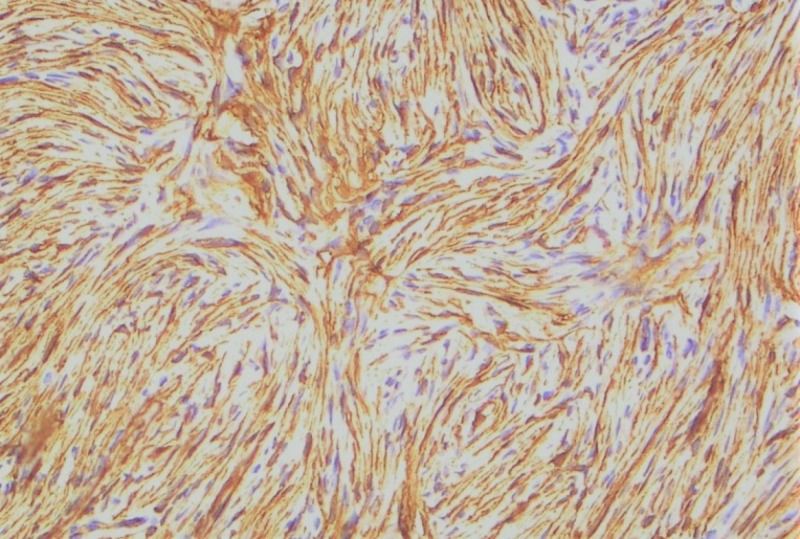
Immunohistochemistry showing intense CD34 positivity in dermatofibrosarcoma protuberans

Two years after the second recurrence, the disease recurred in the left lower limb stump. At this point, a re-biopsy was done and DFSP was confirmed (Figure 1). There was no fibrosarcomatous transformation. Immunohistochemistry was positive for CD34 (Figure 2), focally positive for actin, and negative for desmin and S100 protein. Metastatic workup, consisting of chest X-ray and abdominal ultrasound, was negative. He commenced imatinib mesylate at a recommended dose of 400 mg twice daily. The recurrent lesion on the left lower limb stump gradually progressed in size after five months of imatinib mesylate treatment, and he eventually developed multiple lesions on the posterior torso.

He was then referred to the National Radiotherapy Oncology and Nuclear Medicine Centre, Korle-Bu Teaching Hospital in Accra. Physical examination showed a 15 x 12 cm mobile mass on the left lower limb stump and two palpable, firm, and fixed lesions on the posterior torso; the largest measuring 9 x 7 cm in size. There was no other clinical or radiological evidence of distance metastases. The decision was made to treat him with palliative radiotherapy to relieve pain in the left lower limb stump and to enable him to use the prosthetic limb.

Six months after stopping imatinib mesylate, he received conformal radiotherapy with Cobalt-60 to the left lower limb stump; 40 Gy in 20 fractions, five fractions a week, which he tolerated very well. On a regular five months post-radiotherapy follow-up, there was no palpable or visible lesion on the left lower limb stump or the posterior torso. At 12 months post last follow-up and 17-month post palliative radiotherapy, there was no clinical or radiological evidence of local or distant metastases.

## Discussion

Dermatofibrosarcoma protuberans

Dermatofibrosarcoma protuberans (DFSP) is a rare, indolent cutaneous sarcoma, with a high rate of local recurrence, which rarely metastasizes. It occasionally undergoes a fibrosarcomatous (FS) transformation, which is more aggressive and is associated with a high risk of distant metastasis [8]. DFSP is characterized by the translocation of chromosomes 17 and 22, which causes the formation of a ring chromosome and gene fusion of collagen type 1A1 (COL1A1) and platelet-derived growth factor β (PDGFβ). The COL1A1- PDGFβ fusion gene activates autocrine signaling of PDGF receptor β (PDGFRβ), a tyrosine kinase, for normally inhibited PDGFβ to be activated [9]. DFSP is mostly positive for CD34 and negative for S100 and desmin. Surgical resection with an adequate resection margin is the optimal treatment for localized DFSP. Adjuvant radiotherapy is indicated in close or positive surgical margins and large and unresectable tumors. Imatinib mesylate (IM), a PDGFRβ inhibitor, has shown appreciable response rates for advanced DFSP patients, with a few achieving complete clinical remission [10].

Radiation-induced immune response

Radiotherapy (RT) remains an important and cost-effective cancer treatment option in achieving local tumor control, which translates into improved survival and provides sustained symptomatic relief. These localized effects are achieved through modes of cell death, such as apoptosis, necrosis, mitotic catastrophe, autophagy, or replicative senescence [11]. In recent years, the systemic effect of localized radiotherapy, the abscopal effect, has been reported, mostly occurring concurrently or sequentially with other therapies but rarely with radiotherapy alone.

With the advent of immunotherapy that blocks antibodies to cytotoxic T-lymphocyte-associated protein 4 (CTLA4), programmed cell death-1 (PD-1) and programmed death-ligand 1 (PDL-1), the abscopal effect of radiotherapy is being reported more frequently [11]. The radiation-induced systemic anti-tumor effect is thought to be mediated through the immune system, leading to immunogenic cell death (ICD) [4]. Evidence shows that for ICD to manifest, radiotherapy induces epitope spreading, a phenomenon where self-antigens released from damaged tumor cells prime tumor-specific T cells, which causes further tumor cell damage for more self-antigens to be released and the priming of tumor-specific T cells to be upregulated [12]. Radiotherapy may also aid in the priming of the tumor-specific T cells; a process that requires antigen presentation by matured dendritic cells (DC). Three definite molecular signals are instrumental in achieving ICD in tumors [13]: the pre-apoptotic translocation of calreticulin (CRT) to the cell surface, the release of adenosine triphosphate (ATP) during the blebbing phase of apoptosis, and the cell-death-associated release of high mobility group box 1 (HMGB1). These molecular signals act to boost the DC phagocytosis of tumor cells, the processing and presentation of antigens, and the priming of tumor-specific T cells. The radiation dose at which the immune-stimulatory effect of radiotherapy occurs is still being investigated.

Despite the immune-stimulatory effect of radiotherapy, the immunosuppressive effect is a common finding in RT treatment. RT produces immunosuppressive cytokines and matrix metalloproteinase and myeloid cells, such as myeloid-derived suppressor cells, which together with regulatory T cells effectively suppress the anti-tumor effects of tumor-specific T cells [14]. Inappropriate antigen presentation or activation signaling from the DC can also lead to the immunosuppressive effect. The ability of radiotherapy to stimulate and inhibit the immune system is a likely explanation of why the abscopal effect rarely occurs in treatment with radiotherapy alone.

Adaptive immunity of imatinib

Imatinib mesylate (IM) is a small molecule tyrosine kinase inhibitor of breakpoint cluster region-Abelson (BCR-ABL) fusion protein, KIT and PDGFR. It has altered dramatically the mode of treatment of some malignancies. It is used to effectively treat chronic myeloid leukemia (CML) by targeting the BCR-ABL fusion protein and gastrointestinal stromal tumors (GISTs) by targeting KIT and platelet-derived growth factor α (PDGFRα). It has also shown some efficacy in advanced DFSP through the targeting of PDGFRβ. IM has been associated with the direct inhibition of tumor growth and p16, p21, p53-induced senescence in sarcoma models [15]. For senescent cells to progress to apoptotic death, tumor-specific T cells play an essential role. In recent years, IM-induced immune response has been reported in GIST, for which natural killer (NK) cells played an important role [6,16]. Further studies revealed that multiple immune and inflammatory-related pathways are upregulated in DFSP patients treated with IM. These pathways are marked by antigen presentation, cytotoxic T lymphocytes (CTLs)-mediated apoptosis of targeted cells, and interferon signaling [15].

Despite its success, resistance to IM usually occurs. Interferon-gamma induces the human leukocyte antigen (HLA) class I molecule that is essential in the antigen presentation pathway and the expression of PDL1 [17]. The expressed PDL1 is known to cause the anergy and deletion of tumor-reactive cells [18], which contributes to IM resistance. CTLs and NK cells can downregulate the death receptor that may prevent ligand-mediated tumor death [19] and possibly cause IM resistance. Another likely contributor to IM resistance is selective therapeutic pressure that causes IM-sensitive cells to be killed leaving the resistant cells.

Concurrent imatinib and radiotherapy

There are currently few reported cases showing the clinical benefits of concurrent IM and radiotherapy for which lower radiation doses have been used to achieve desirable outcomes [7]. In a preclinical study of muscle-invasive bladder cancer, IM acted as a radiosensitizer by targeting homologous recombination [20]. In the study, IM was associated with reduced RAD51 expression, with and without ionizing radiation, and there was no radiosensitivity effect in bladder cancer Ku knockdown (Ku80KD) cells.

Effect of the timing sequence 

A major factor that may play a role in achieving the abscopal effect is the timing of treatment, i.e., if IM should be given prior to radiotherapy for adequate senescent cells to be generated or it should be given concurrently with radiotherapy. IM induces the expression of PDL1, which may increase sensitivity to immunotherapy. Combining IM with both immunotherapy and radiotherapy will possibly modulate the systemic anti-tumor effect of radiotherapy that is likely to be produced by concurrent or sequential treatment with immunotherapy and radiotherapy alone.

Limitation

The metastatic lesions were not histopathologically confirmed. They were clinically diagnosed based on the fact that they first appeared when IM resistance by the primary tumor was first observed. In addition, the clinical features were consistent with a tumor as follows: the lesions were non-tender, slow growing, a firm and lumpy characteristic on physical examination, and clinically regressed with the primary lesion, after the primary lesion was irradiated. It would have been prudent to demonstrate the observation with images but given the retrospective nature of this report, this is understandable.

## Conclusions

The case report discussed the demonstrated abscopal effect of localized radiotherapy following resistance to IM therapy in a 56-year-old male with metastatic DFSP. The exact role of immune response to IM in the treatment of DFSP is yet to be determined. NK cells, predominantly, and tumor-specific T cells have been associated with IM-induced cell death, and their downregulation may lead to IM resistance. Radiotherapy may play a role in upregulating tumor-specific T cells. Therefore, by combining IM, which induces tumor senescence, and RT, which upregulates the tumor-specific T cells needed for causing apoptotic cell death from senescent cells, the abscopal effect of radiotherapy can be enhanced.
